# Advanced Doppler Ultrasound Insights: A Multicenter Prospective Study on Healthy Skin

**DOI:** 10.3390/diagnostics15050569

**Published:** 2025-02-26

**Authors:** Priscila Giavedoni, Jorge Romaní, Francisco de Cabo, Francisco Javier García-Martínez, Mónica Quintana-Codina, Esther Roè-Crespo, Irene Fuertes de Vega, Xavier Soria-Gili, Rafael Aguayo-Ortiz, Patricia Garbayo-Salmons, Gonzalo Castillo, David Vidal-Sarró, Jordi Mollet, Laura Serra, Carlos Gonzalez, Emilio López-Trujillo, Miquel Just, Marc Combalia, Sebastian Podlipnik, Josep Malvehy, Ximena Wortsman

**Affiliations:** 1 Dermatology Department, Hospital Clínic Barcelona, 08036 Barcelona, Spain; ifuertes@clinic.cat (I.F.d.V.); lserra@clinic.cat (L.S.); marccombalia@gmail.com (M.C.); spodlipnik@gmail.com (S.P.); jmalvehy@gmail.com (J.M.); 2 Dermatology Department, Hospital Granollers, 08402 Granollers, Spain; jromani@tauli.cat; 3 Ultrasound Department, Instituts Guirado Radiolgia, 08022 Barcelona, Spain; 4 Department of Dermatology, Clínica Dermatológica Internacional, Department of Medicine, Faculty of Health and Sports Science, Universidad Europea de Madrid, 28670 Villaviciosa de Odón, Spain; fjgarcia@aedv.es; 5 Dermatology Department, Hospital Universitari Sagrat Cor, Grupo Quironsalud, 08029 Barcelona, Spain; mquintanacodina@gmail.com; 6 Dermatology Department, Hospital Sant Pau I la Santa Creu, 08025 Barcelona, Spain; esther.roecrespo@gmail.com; 7 Dermatology Department, Hospital Universitari Arnau de Vilanova de Lleida, 25198 Lleida, Spain; soriaxavier@gmail.com (X.S.-G.); aguayolleida@gmail.com (R.A.-O.); 8 Dermatology Department, Hospital Mutua de Terrassa, 08221 Terrassa, Spain; pgarbayo@gmail.com; 9 Dermatology Department, Hospital Universitario Germans Trias y Pujol de Barcelona, 08916 Badalona, Spain; gonzalocastillocapponi@gmail.com; 10 Dermatology Department, Hospital Universitari d’Igualada, 08700 Barcelona, Spain; david.vidal.sarro@gmail.com (D.V.-S.); jmollet@vhebron.net (J.M.); 11 Dermatology Department, Hospital Vall d’Hebron, 08035 Barcelona, Spain; dermatologia.carlos.gonzalez.cruz@gmail.com; 12 Dermatology Department, Hospital Mar Parc de Salut de Barcelona, 08003 Barcelona, Spain; emilio08tr@gmail.com; 13 Dermatology Department, Hospital Universitari Joan XXIIII de Tarragona, 43005 Tarragona, Spain; 14 Department of Dermatology, Faculty of Medicine, Universidad de Chile, Santiago 8380453, Chile; xworts@yahoo.com; 15 Institute for Diagnostic Imaging and Research of the Skin and Soft Tissues (IDIEP), Santiago 8380453, Chile

**Keywords:** cutaneous Doppler ultrasound, healthy skin, visible vessels, spectral Doppler, color Doppler, power Doppler

## Abstract

**Background:** There have been multiple studies on the use of Doppler ultrasound to define skin inflammation, but the visible vessels of healthy skin have yet to be described. **Objective:** This study aimed to evaluate the visible vessels of healthy skin using Doppler ultrasound. **Methods:** Prospective multicenter study using Doppler ultrasound to analyze healthy skin. The color percentage, flow velocity, and maximum vessel diameter were calculated. **Results:** 943 images from 152 patients were recorded. The most frequently used mode was color Doppler (40.6%), followed by power Doppler (30.4%). Visible vessels were detected in 18.23%; in positive Doppler images, color occupied less than 5%. The malar region exhibited the highest visible vessels. The 22 MHz probe detected smaller vessels with slower flows than the 18 MHz probe. Spectral Doppler showed peak systolic values of less than 10 cm/s and a vessel diameter of less than 1 mm. In most of the participating centers, the operators had less than 10 years of experience in performing skin ultrasound examinations. Sensitivity of the Doppler may vary according to the device. **Conclusions:** With the used ultrasound equipment, it was uncommon to visualize vessels in healthy skin. When seen, they covered less than 5% of the image with low flow and small size.

## 1. Introduction

Doppler ultrasound is a noninvasive technique used in dermatology to evaluate visible vessels in inflammatory diseases, the etiology and evolutionary stage of vascular lesions, and visible vessels in skin tumors [1]. It also makes it possible to precisely locate vascular structures before surgery, thus guaranteeing safety margins [2]. Although numerous studies have been performed using Doppler ultrasound to detect inflammation and increased visible vessels in tumors, surprisingly, there is no established Doppler color pattern for healthy skin. Furthermore, variability in the visualization of blood vessels resulting from the different equipment and probes used in Doppler ultrasound examinations could modify the Doppler color pattern considered “normal” for healthy skin. In this multicenter study, we describe the Doppler ultrasound patterns of the visible vessels of healthy skin with the high-frequency ultrasound equipment currently used in dermatology.

## 2. Materials and Methods

### 2.1. Study Design

A multicenter cross-sectional study was conducted, between 1 August 2021 and 30 November 2021, to evaluate healthy skin Doppler images using high-frequency ultrasound equipment with 18 and 22 MHz probes.

All operators involved in this study had a minimum of one year of experience using high-frequency ultrasound for dermatological purposes. Additionally, each operator performed at least 300 skin ultrasounds annually, meeting the criteria established by the DERMUS Group in their consensus document “*Proposal for an Assessment Training Program in Dermatologic Ultrasound*”. According to this guideline, performing 300 high-frequency skin ultrasounds per year qualifies an operator as an expert. This ensures that the images obtained and their interpretation in this study were conducted by professionals with sufficient expertise.

The following variables were determined and assessed: the primary variable was blood flow in healthy skin with the Doppler mode; secondary variables were age, sex, ethnicity, comorbidities, the reason for the dermatological consultation vs. healthy volunteers, skin type, and evidence of sun damage. In addition, patient and room temperature, smoking habits, and medication intake were also recorded. The study was reviewed and approved by the ethics committee of Hospital Clínic de Barcelona (Reg. HCB/2020/1460) and subsequently at each of the hospitals where the study was performed.

### 2.2. Ultrasound Evaluation

Color Doppler, power Doppler, or X-Flow modes were used to visualize the small vessels of the dermis and subcutaneous tissue. Doppler measurements were made with an insonation angle equal to or less than 60°. The pulse repetition frequency was fixed at 750 Hz. The color gain was variable and adjusted to the value immediately below the noise threshold. These settings were standardized across operators to ensure consistency in the images obtained. Six zones were studied in each participant: the nasolabial fold, malar region, the middle third of the dorsum of the arm, the flank, the middle third of the inner thigh, and the infraclavicular cleavage.

The images taken always included the dermis and subcutaneous tissue while avoiding muscle. The depth of the images varied depending on the individual and the location under study. When Doppler flow was seen, three images were analyzed: the image with the most significant number of vessels visualized with color Doppler; the image with the largest arterial vessel diameter (the largest arterial vessel was measured in mm); and the image showing the Doppler spectral values of the arterial vessel with the fastest flow, measured in cm/s.

### 2.3. Study Population

The inclusion criteria were patients with a skin pathology limited to an anatomical area other than where the measurements were taken, healthy volunteers who were relatives of patients, and students or healthy workers at the participating centers. It was a requirement that patients had not eaten, exercised, or consumed alcohol for at least 4 h before the study. Participants or guardians of minors who could understand and comply with the study requirements signed an informed consent form after receiving information on the study design, objectives, and potential risks. Exclusion criteria included inflammatory diseases, infectious diseases, or malignant tumors that could affect the evaluation sites or determine a state of systemic vasodilatation or vasoconstriction, as they could affect the visible vessels of apparently healthy skin. Children under 4 years were also excluded, as sedation is often necessary for imaging this age group, which can modify cutaneous visible vessels.

### 2.4. Duration of the Study

The study was conducted for a maximum period of 4 weeks. Each patient was attended once, and the ultrasound study lasted between 15 and 30 min.

### 2.5. Statistical Analysis

Pearson’s X^2^ and trend tests for ordinal variables were used to compare categorical and ordinal variables, respectively. For continuous paired variables, the Wilcoxon test was used to compare two groups of samples, and the Kruskal–Wallis test was used to compare multiple groups. The analysis used the computing environment R and RStudio and used a two-sided type I error of 0.05.

## 3. Results

Thirteen Spanish centers participated in the study. Eleven dermatologists and one radiologist performed the imaging, all of whom specialized in skin ultrasound. In addition, an international reference radiologist in Doppler ultrasound collaborated to validate the correction of the images before analysis. The experience of the operators was variable; 41.6% (*n* = 5) had more than 5 years of experience in cutaneous Doppler ultrasound, and the remainder (*n* = 7) were trainees with less than 5 years of experience. The ultrasound instruments used were Canon Aplio A (*n* = 2), Esaote My Lab Class C (*n* = 1), Esaote My Lab Gamma (*n* = 5), Esaote My Lab Seven (*n* = 1), Esaote My Lab 25 Gold (*n* = 2), and General Electric Logiq-P9 (*n* = 1). A total of 152 patients were included, and 943 images were generated; 366 images were acquired with 18 MHz probes and 577 images with 22 MHz probes.

Blood flow could be measured with spectral Doppler in 172 of the 883 images realized, since Doppler flow was only visualized in 18.23% of the images taken. In the images with Doppler flow, the proportion of color in the total image was less than 5%.

The area where the vessels were visualized differed according to the probe used.

With the 18 MHz probe, basal areas were visualized predominantly in the hypodermis. The distribution was as follows: In the nasolabial fold, 6.9% of the localized vessels were visualized in the dermis, 69% in the hypodermis, and 24.1% in both areas. In the malar area, 8% of the vessels were visualized in the dermis, 72% in the hypodermis, and 20% in both areas. In the flank, 3.8% of the vessels were visualized in the dermis, 90.4% in the hypodermis, and 5.8% in both areas. In the thigh, 87.9% of the vessels were visualized in the hypodermis and 12.1% in both areas (dermis and hypodermis). No vessels were found only in the dermis on the neckline, although 95.5% were seen in the hypodermis and 4.5% in both areas. In the arm, 2.3% of the vessels were observed in the dermis, 97.7% in the hypodermis, and none in both areas.

With the 22 MHz probe, vessels in the dermis were predominantly visualized. In the nasolabial fold, 34.3% of the vessels were observed in the dermis, in the hypodermis 15.2%, and in both areas 50.3%. In the malar area, 36.4% of the vessels were observed in the dermis, in the hypodermis 26.3%, and in both areas 37.4%. In the flank, 21% of the vessels were observed in the dermis, in the hypodermis 41%, and in both areas 38%. In the thigh, 22.2% of the vessels were observed in the dermis, in the hypodermis 56.7%, and in both areas 21.1%. The 32.3% of the neckline vessels were observed in the dermis, in the hypodermis 45.2%, and in both areas 22.6%. On the upper arm, 31.1% of the vessels were observed in the dermis, in the hypodermis 48.9%, and in both areas 20%.

**Figure 1:**
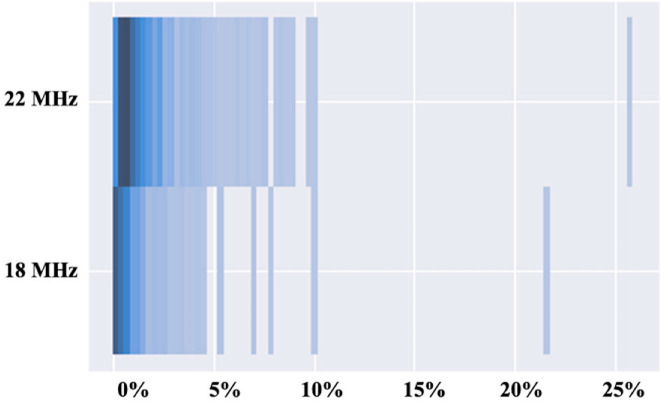
Distribution of Doppler percentages—this figure illustrates the varying percentages of Doppler signals detected across different skin regions using both the 18 MHz and 22 MHz evaluation probes.

Table 1 shows the characteristics of the physicians and patients. Figure 1 shows the percentage occupied by color in the image, with the Doppler mode in the 18 MHz and 22 MHz probes. Vessels were observed more frequently with 22 MHz probes than 18 MHz probes. The 22 MHz probe detected smaller vessels with slower flows, while the 18 MHz probe detected larger vessels with faster flows. Vessels in the dermis were more easily detected with 22 MHz probes than with 18 MHz probes.

**Figure 2:**
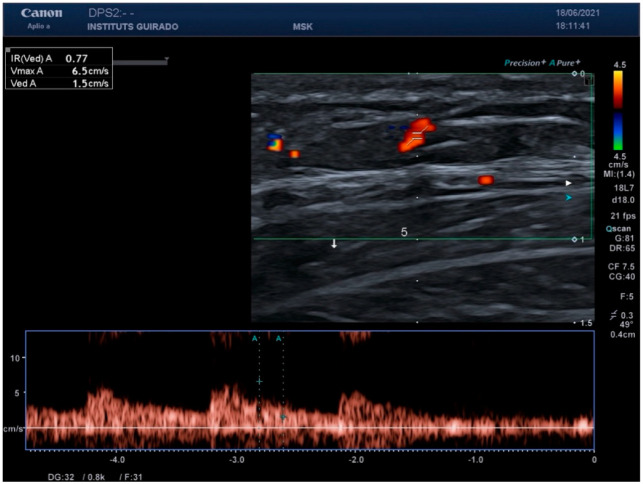
Spectral Doppler analysis—a spectral Doppler image depicting an arterial vessel in healthy skin, showcasing typical flow characteristics and peak systolic values.

**Figure 3:**
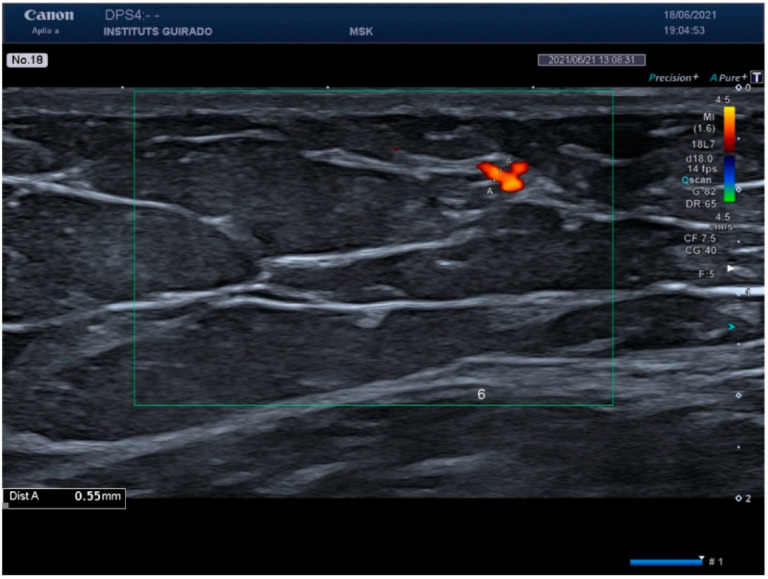
Vessel diameter measurement—this figure presents the measurement of vessel diameters in healthy skin, highlighting the detection capabilities of the Doppler ultrasound.

When analyzing the spectral Doppler results, peak systolic values of less than 10 cm/s were found in the six areas studied. Regarding vessel diameter, healthy skin vessels were smaller than 1 mm in all areas and with both probes. Figure 2 shows the spectral Doppler of an arterial vessel in healthy skin, and Figure 3 shows the vessel diameter.

Table 2 shows the Doppler values of all the areas studied with the different probes.

## 4. Discussion

High-frequency skin ultrasound (HFUS) is a relatively new technique in dermatology [3]. It has the advantage of visualizing the different layers of the skin in detail and is noninvasive and inexpensive. Its use has grown significantly over the past decade, leading to the identification of numerous skin conditions through HFUS. [4,5]. Thus, HFUS is considered a complementary technique to clinical examination findings. It is now widely applied for diagnosis, follow-up, and treatment decisions in various skin pathologies [6,7].

Imaging of healthy skin in B-mode is well established and is characterized by fine hyperechogenic lines in the epidermis and muscle fascia. The dermis is hyperechogenic compared to the hypodermis. The hypodermis has hypoechoic lobules and thin septa that resemble hyperechogenic moons. The muscle is hypoechoic, with fine parallel hyperechogenic lines forming a fibrillar pattern, which distinguishes it from the hypodermis [8]. However, the visible vessels of healthy skin as they appear with the Doppler mode have not yet been well-documented, despite long-established findings on Doppler modes in inflamed skin. Therefore, it is necessary to determine the Doppler pattern of healthy skin to provide a contrast to inflamed skin.

The Doppler mode is used to study the velocity of fluids, especially blood. There are three key Doppler modes: (1) Color Doppler, which allows the flow direction to be studied. It provides both qualitative estimates of blood velocity and quantitative data, such as the location, number, and diameter of vessels; (2) Power Doppler, which is more sensitive than color Doppler for visualizing low-velocity vessels, such as those in the skin. The disadvantage of power Doppler is that it does not usually indicate flow direction and cannot estimate velocity; (3) Pulsed Doppler, which is used to determine whether a vessel is arterial, venous, or an arteriovenous fistula and to measure flow velocity quantitatively [1,9].

In our study, we found that visualizing vessels in healthy skin is challenging with the HFUS devices used. When vessels were visible, they occupied less than 5% of the total image. Furthermore, we determined that arterial velocity in the arterial vessels of healthy skin is less than 10 cm/s. These vessels were typically thin, with diameters below 1 mm. However, the devices used in the study corresponded to medium-gamma ultrasound equipment, indicating that more advanced devices might improve sensitivity to detect low-flow velocities in the skin.

Since HFUS began to be used in dermatology, numerous studies have characterized inflammation patterns and vascular structures in tumors and vascular pathologies, aiding in diagnosis and treatment monitoring [10,11,12]. In cutaneous oncology, HFUS allows us to explore the etiology of the tumor. For benign tumors, HFUS is often sufficient for diagnosis, making it useful for identifying conditions such as epidermoid cysts, pilomatrixomas, lipomas, and osteomas [9]. Hemangiomas and arteriovenous and lymphatic malformations also have characteristic ultrasound patterns, primarily observed in the Doppler mode [13,14]. In malignant tumors, HFUS contributes to diagnosis in both the B-mode and Doppler mode. For example, basal cell carcinoma often presents with distinctive hyperechogenic spots in B-mode [15]. In contrast, squamous cell carcinoma, melanoma, and dermatofibrosarcoma protuberans typically show markedly increased Doppler flow in vertical vessels [16,17,18].

Inflammatory skin diseases are commonly evaluated through physical examination and, in many cases, skin biopsy to assess histological features. Advanced imaging techniques such as magnetic resonance imaging and computed tomography are used sparingly in dermatology due to their limited resolution for skin layers [9]. Moreover, these techniques require contrast, are costly, and are only accessible in specialized centers. Dermoscopy, confocal microscopy, and optical coherence tomography are increasingly utilized but are limited to studying the superficial dermis [19,20,21]. In contrast, HFUS is an excellent technique for evaluating inflammatory diseases, with the color Doppler mode playing a critical role in determining disease activity and assessing whether inflammation involves the dermis, hypodermis, fascia, or muscle [9]. Additionally, HFUS identifies the most inflamed site, enabling targeted biopsies for optimal diagnostic yield [22].

In patients with autoimmune and systemic inflammatory diseases, HFUS quantifies inflammation and measures skin thickening during active phases and atrophy during residual stages [23,24,25]. This objective disease activity assessment facilitates safer and more tailored therapeutic decisions. Previous studies have described Doppler parameters for inflammation in conditions such as autoimmune panniculitis, graft-versus-host disease, morphea, and systemic sclerosis [25,26]. However, most studies have relied on subjective assessments of increased color Doppler signals to define inflammation, underscoring the need for standardized reference values. Our findings for healthy skin—such as the percentage of color Doppler signals, flow velocity, and vessel diameter—provide a foundation for detecting inflammation.

In diseases like hidradenitis suppurativa, where staging is complex, HFUS improves patient evaluation [27,28,29]. The ultrasound-adapted Hurley scale has demonstrated greater sensitivity than clinical staging, as it detects subclinical lesions [30]. Doppler findings in hidradenitis suppurativa also correlate with histological results, though current descriptions are largely qualitative.

Many studies using the color Doppler mode associate swelling with “increased vascularity”, but this term is imprecise because healthy skin also exhibits detectable Doppler flow. Quantifying vascularity differentiates normal blood flow from inflammation by measuring vessel density, flow velocity, and diameter. Few published studies include spectral Doppler parameters, making it difficult to compare and validate findings for healthy skin.

In conclusion, our results establish a baseline for defining inflammation using HFUS. Measuring the visible vessels of healthy skin with current ultrasound technology is a crucial step toward improving diagnostic accuracy for skin diseases.

## 5. Limitations

The number of patients included in the study was limited. Additionally, the operators’ experience was less than 10 years in most of the participating centers, which might have influenced the findings. Patients were assessed with either 18 MHz or 22 MHz probes, but no assessments were conducted using both probe types on the same patients. Medium-gamma ultrasound devices were used to assess normal skin vascularity. This could limit the detection of subtle vascular patterns, suggesting the need for validation with high-end devices. Finally, six different models of ultrasound equipment were used; therefore, our results require further validation using other commonly available equipment, particularly those representing lower- and higher-end devices.

## Figures and Tables

**Table 1:** Characteristics of the sonographers and patients.

Ultrasound Technician Expertise	
In training	7 (58.4%)
Expert	5 (41.6%)
**Years of experience**	4.0 (3.0–7.0)
**Reason for Dermatology visit**	
Skin disease	1 (0.7%)
Healthy volunteer	151 (99%)
**Axillary temperature °C**	36.20 (35.95–36.50)
**Room temperature °C**	24 (23–24)
**Gender**	
Female	107 (70%)
Male	45 (30%)
**Weight**	63 (55–73)
**Age**	35 (27–52)
**Ethnic**	
African American	3 (2.0%)
Caucasians	149 (98%)
**Phototype**	
I	6 (4.0%)
II	40 (27%)
III	85 (57%)
IV	17 (11%)
V	1 (0.7%)
**Smokers (packs/year)**	
0	126 (83%)
0–10	20 (13%)
10–20	2 (1.3%)
>20	4 (2.6%)
**Actinic damage**	
I	117 (77%)
II	30 (20%)
III	5 (3%)
**No pathological history**	139 (91.4%)
**Arterial hypertension**	11 (7.2%)
**Type II diabetes mellitus**	1 (0.7%)
**Chronic renal failure**	1 (0.7%)

**Table 2:** Doppler values of all the areas studied with the different probes.

	18 MHz Probe *N* = 67	22 MHz Probe *N* = 105
**Doppler Type**		
Color	35 (52%)	71 (68%)
Power	32 (48%)	34 (32%)
**Nasolabial fold**		
Peak systolic (cm/s)	6.5 (5.7–8.8)	4.6 (3.5–6.8)
Resistance index	0.71 (0.67–0.74)	0.68 (0.59–0.75)
Vessel diameter (mm)	0.78 (0.58–0.99)	0.52 (0.37–0.78)
**Malar region**		
Peak systolic (cm/s)	2.80 (1.55–3.30)	1.60 (0.90–2.60)
Resistance index	0.73 (0.68–0.75)	0.70 (0.60–0.80)
Vessel diameter (mm)	0.53 (0.45–0.59)	0.46 (0.36–0.65)
**Flank**		
Peak systolic (cm/s)	8.4 (6.8–9.5)	4.7 (3.7–6.2)
Resistance index	0.70 (0.65–0.74)	0.67 (0.60–0.80)
Vessel diameter (mm)	0.78 (0.44–0.95)	0.48 (0.34–0.67)
**Thigh**		
Peak systolic (cm/s)	8.2 (6.4–9.9)	5.0 (4.1–6.4)
Resistance index	0.73 (0.69–0.80)	0.68 (0.58–0.73)
Vessel diameter (mm)	0.66 (0.52–0.81)	0.53 (0.38–0.77)
**Neckline**		
Peak systolic (cm/s)	6.6 (5.5–7.6)	4.2 (3.2–6.3)
Resistance index	0.75 (0.70–0.78)	0.68 (0.60–0.80)
Vessel diameter (mm)	0.55 (0.47–0.94)	0.47 (0.37–0.61)
**Arm**		
Peak systolic (cm/s)	6.30 (4.30–7.45)	3.70 (2.55–5.03)
Resistance index	0.71 (0.69–0.73)	0.70 (0.61–0.76)
Vessel diameter (mm)	0.64 (0.43–0.85)	0.42 (0.33–0.59)

## Data Availability

The original contributions presented in this study are included in the article. Further inquiries can be directed to the corresponding author.
